# The Impact of Clinical Factors and SARS-CoV-2 Variants on Antibody Production in Vaccinated German Healthcare Professionals Infected Either with the Delta or the Omicron Variant

**DOI:** 10.3390/vaccines12020163

**Published:** 2024-02-05

**Authors:** Catharina Gerhards, Marlene Steingass, Alexandra Heininger, Bettina Lange, Michael Hetjens, Marlis Gerigk, Michael Neumaier, Osman Evliyaoglu, Maximilian Kittel

**Affiliations:** 1 Institute for Clinical Chemistry, University Medical Center Mannheim, Medical Faculty Mannheim, University of Heidelberg, 68167 Mannheim, Germany; 2 Department of Hygiene, University Medical Center Mannheim, Medical Faculty Mannheim, University of Heidelberg, 68167 Mannheim, Germany; 3 Department of Biomedical Informatics, Center for Preventive Medicine and Digital Health Baden-Württemberg, University Medical Center Mannheim, Medical Faculty Mannheim, Heidelberg of University, 68167 Mannheim, Germany; 4 Institute of Medical Microbiology and Hygiene, University Medical Center Mannheim, Medical Faculty Mannheim, University of Heidelberg, 68167 Mannheim, Germany

**Keywords:** anti-SARS-CoV-2 antibodies, anti-N abs, anti-S1/RBS, SARS-CoV-2 vaccination, COVID-19 after vaccination, vaccination breakthrough, serological immune response

## Abstract

Background: The aim of the rapid introduction of vaccines during the COVID-19 pandemic was a reduction in SARS-CoV-2 transmission and a less frequent occurrence of severe COVID-19 courses. Thus, we evaluated COVID-19 severity in vaccinated individuals to examine variant-specific symptom characteristics and their clinical impact on the serological immune response. Methods: A total of 185 individuals previously vaccinated against and infected with the SARS-CoV-2 Delta (B.1.617.2) or Omicron (BA.4 and BA.5) variant, were enrolled for anti-SARS-CoV-2 anti-N- and anti-RBD/S1-Ig level detection. A structured survey regarding medical history was conducted. Results: In 99.5 percent of cases, outpatient treatment was satisfactory. Specific symptoms associated with variants included ageusia and anosmia in patients with Delta infections and throat pain in Omicron infections. Among Delta-infected individuals with specific symptoms, significantly higher levels of anti-N antibodies were observed. Conclusion: Our study identified variant-specific differences in the amount of SARS-CoV-2 antibody production and COVID-19 symptoms. Despite this, vaccinated individuals with Omicron or Delta infections generally experienced mild disease courses. Additionally, asymptomatic individuals exhibit lower anti-SARS-CoV-2 antibody levels, indicating a clinical correlation between disease-specific antibodies and distinct symptoms, particularly in the case of the Delta variant. In follow-up studies, exploring post-COVID syndrome and focusing on cognitive symptoms in the acute phase of Omicron infections is crucial as it has the potential to longitudinally impact the lives of those affected.

## 1. Introduction

As the COVID-19 pandemic has burdened the global healthcare system with high hospitalization rates, the implementation of vaccines aimed to reduce SARS-CoV-2 transmission, serious COVID-19 courses, and general symptom severity [1,2]. Accordingly, the humoral immune response could be stimulated by various vaccines available on the market such as messenger RNA (mRNA)-based products including BNT162b2 (Comirnaty, Pfizer-BioNTech, Mainz, Germany) and mRNA-1273 (Spikevax, Moderna, Cambridge, USA) or vector-based products such as ChAdOx1 nCoV-19 (AstraZeneca, Cambridge, UK) and Ad16.COV.2.S (Janssen Pharmaceutical K.K, Beerse, Belgium) [3,4,5]. The underlying mechanism of vaccination is to stimulate the recipient’s immune system to produce disease-specific antibodies without having to experience the disease themselves. These antibodies can prevent infection or reduce the extent of systemic spread in the event of exposure to the pathogen. Here, we are referring to anti-SARS-CoV-2 antibodies targeting the Spike (S) or Receptor-Binding Domain (RBD). In contrast, the detection of anti-Nucleocapsid (N) antibodies indicates a previous infection, as no vaccine was included in the study cohort that featured the nuclear capsid as an antigen target structure [6,7]. This may lead to an unpredictable response from individuals who experience a less severe course of COVID-19 due to previous vaccination. It suggests the possibility that the immune response specific to the infection might be more challenging to detect, potentially because of reduced viral dissemination. This is an aspect we intend to explore within the scope of this study.

Moreover, we observed an emerging vaccine breakthrough previously, particularly with more contagious SARS-CoV-2 variants such as Omicron, in a prolonged observation period after vaccination [8]. In this context, the question arises whether higher infection rates are nevertheless associated with a more asymptomatic course. Some studies have already described milder courses with less hospitalization and fewer secondary complications in infections with the Omicron variant. However, this requires a comparison cohort with a variant that is associated with severe courses, such as the Delta variant to exclude a variant-based reduction in symptom severity in vaccinated individuals [9,10,11,12]. 

Hence, in this study, we included German healthcare workers with positive SARS-CoV-2 qRT-PCR results during the Delta and Omicron variant periods following SARS-CoV-2 vaccination. In these cohorts, we aimed to assess COVID-19 severity in vaccinated subjects (aim I), anti-SARS-CoV-2 anti-S/RBD and anti-N abs to investigate whether the serological immune response is influenced by SARS-CoV-2 variants (aim II) or by the presence and characteristics of symptoms (aim III).

## 2. Materials and Methods

### 2.1. Participant Recruitment and Sample Collection

**Figure 1:**
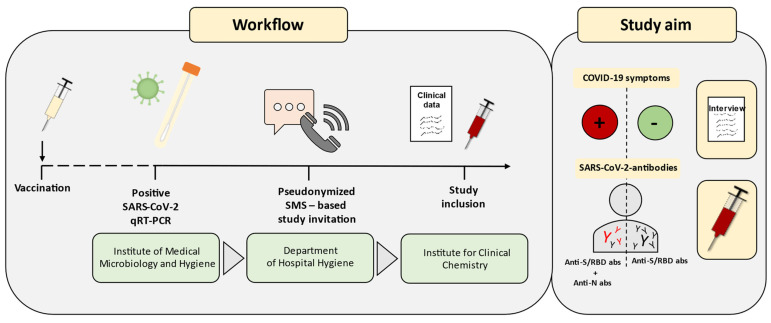
Study workflow and study aims. Vaccinated and recovered COVID-19 subjects were enrolled in the study. SARS-CoV-2 qRT-PCR tests were routinely conducted by the Institute of Medical Microbiology and Hygiene. Positive results were reported to the Department of Hygiene of the University Hospital Mannheim. Utilizing text messages for the report of test results, voluntary participation in the study was offered in this context. Upon acceptance, the Institute of Clinical Chemistry scheduled appointments for study inclusion, involving clinical history and blood sampling. Serological data were assessed, and antibody responses specific to the infection were examined, considering clinic and variant influences. SMS, Short Message Service; abs, antibodies.

**Figure 2:**
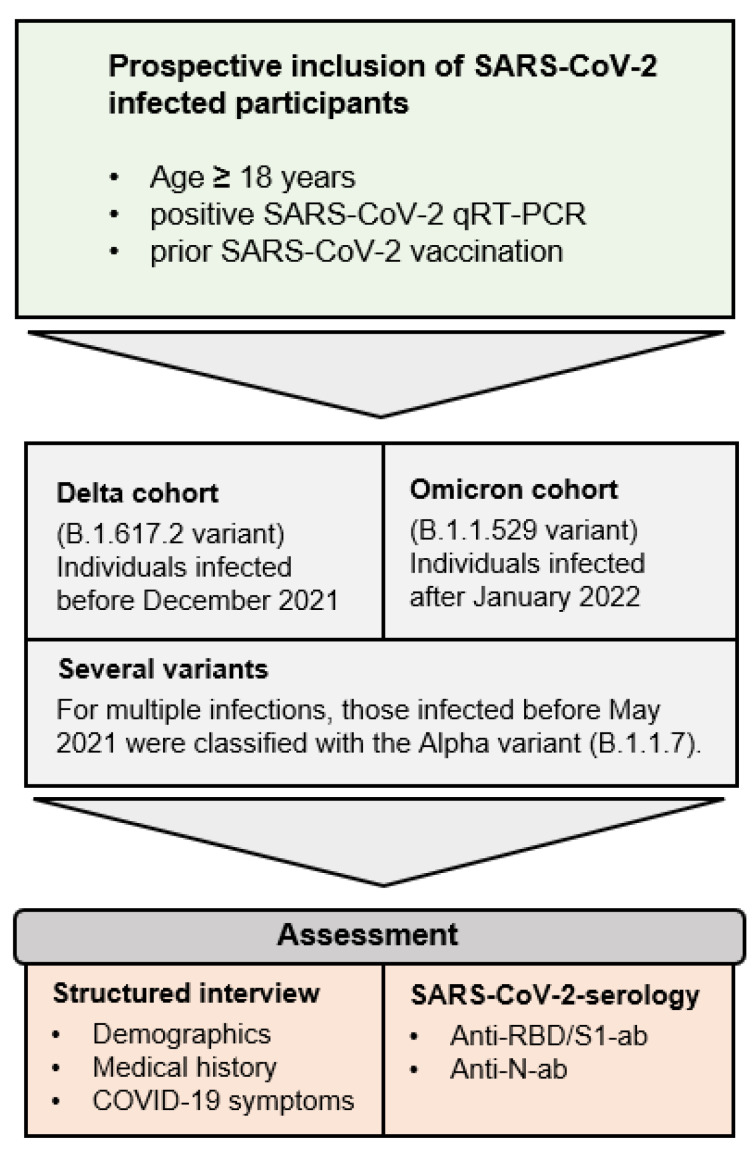
Inclusion criteria and study design.

Adult individuals with positive SARS-CoV-2 qPCR results after prior SARS-CoV-2 vaccination were invited to participate in the Immunitor-IV study at the University Medical Center Mannheim, Germany (Figure 1 and Figure 2). The recruitment phase lasted from April 2022 to June 2022. Given the absence of variant typing for all participants, classification was performed using demographic data from the Robert Koch Institute (RKI). Individuals infected within overlapping time intervals regarding a substantial prevalence of Delta and Omicron, such as December 2021, were excluded from the study. Those who were tested positive for SARS-CoV-2 from 15 January 2022 onward were categorized into the Omicron cohort, while individuals infected before December 2021 were assigned to the Delta cohort. Participants with multiple infections from several variants of concern were analyzed in a separately defined category.

To acquire standardized surveys that capture demographics and medical information, including symptoms of COVID-19 infection and vaccination, we used a secure web platform, Research Electronic Data Capture (REDCap). These structured interviews were not operated by the participants themselves but were conducted and reported by a study leader. We obtained informed consent from all participants, and the study was executed following the guidelines of the Declaration of Helsinki. The Institutional Review Board approved the study under protocol number 2020-556N.

We collected a single blood sample in 7.5 mL lithium heparin tubes (S-Monovette, Sarstedt AG & Co., Nümbrecht, Germany) and centrifuged the samples at 2000× *g* for 10 min at 18 °C, and plasma was aliquoted and stored at −80 °C until analysis.

### 2.2. Anti-SARS-CoV-2 Antibody Detection

As previously described [8], we used an electrochemiluminescence immunoassay (ECLIA) for the qualitative analysis of anti-SARS-CoV-2 anti-N Pan-Ig. This test had CE and FDA approval. Values exceeding the cut-off index (COI) ≥ 1.0 (Elecsys^®^ Anti-SARS-CoV-2 (Roche, Mannheim, Germany)) were evaluated as positive results. In addition, a further assay equally having CE and FDA approval for quantitative measurement of anti-SARS-CoV-2 RBD/S1 antibodies (Elecsys^®^ Anti-SARS-CoV-2 S assay (Roche, Mannheim, Germany)) was used. Reactive outcomes were reported at ≥0.8 U/mL. The methods were previously verified using test controls and patient material according to the DIN EN ISO 15189 [13]. Moreover, we conducted the analyses according to the manufacturer’s instructions at an accredited laboratory.

### 2.3. General Data Analysis

The collection of individuals’ clinical data was performed by use of the REDCap platform (Vanderbilt University, Nashville, TN, USA). Based on Microsoft Excel 2019 (Microsoft, Redmond, WA, USA) and RStudio (version 4.1.2; RStudio, Boston, MA, USA) [14], statistical data evaluation was conducted. In the case of non-normally distributed continuous variables, the Kruskal–Wallis rank sum test was chosen and the Fisher’s exact test served for categorical parameters. If multiple parameters were compared, we augmented the statistical analysis with a Benjamini–Hochberg correction to prevent false-positive results. A significant result exhibited *p*-values below 0.05. The data illustration was realized using RStudio (version 4.1.2; RStudio, Boston, MA, USA) [14] and Microsoft PowerPoint 2019 (Microsoft, Redmond, WA, USA).

## 3. Results

### 3.1. Demographics and Vaccination Strategies

In total, 185 subjects were included in the study, of which 76.8% were female and 23.2% were male. The mean age was 36.04 (+/−11.65); the median BMI was 23.66 (range, 17.7 to 40.1), and 15.1% were smokers. A total of 184 of the participants reported being a medical professional. The majority of respondents reported working in regular care units (35.7%), followed by departments with no contact with patients (30.8%), units with frequent and intensive patient contact in Intensive Care Units (ICUs) (18.9%), and infrequent contact (14.6%). Most subjects were vaccinated with BionTech/Pfizer during their first to third vaccinations. A median of 313 days passed between the first vaccination and the SARS-CoV-2 infection. Twenty-seven individuals were infected before the third vaccination. In participants who were infected after the third vaccination, the median time between positive PCR and the third vaccination was 81 days.

A total of 99.5% of the subjects were treated as outpatients. Only one participant needed hospital admission with normal care treatment. None of the participants needed intensive care treatment, and all participants survived this SARS-CoV-2 infection. A comprehensive medical history regarding the treatment of COVID-19 was not obtained, thereby precluding any statements on potential antiviral therapies, including treatment with immunoglobulins. Further medical aspects as well as pre-existing diseases are summarized in detail in Table 1. The most common symptoms of COVID-19 disease were novel headache (67.0%), followed by rhinitis/runny nose (65.9%), cough (62.2%), and sore throat (62.2%). A more detailed summary of these is given in Table 1 and Table S1.

### 3.2. Anti-SARS-CoV-2 Antibody (ab) Levels

**Figure 3:**
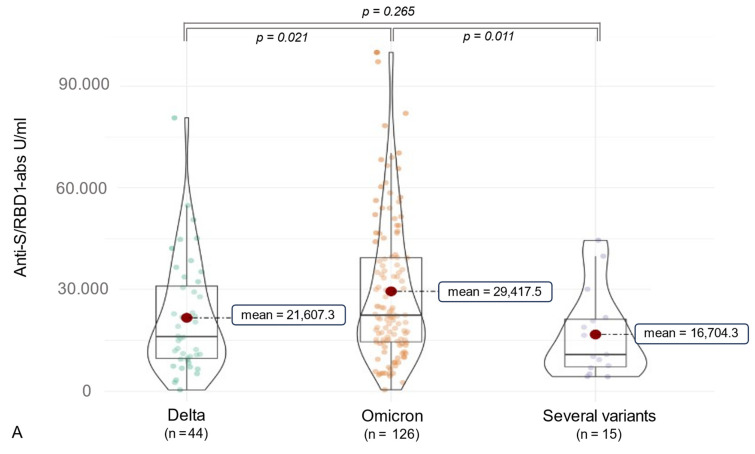

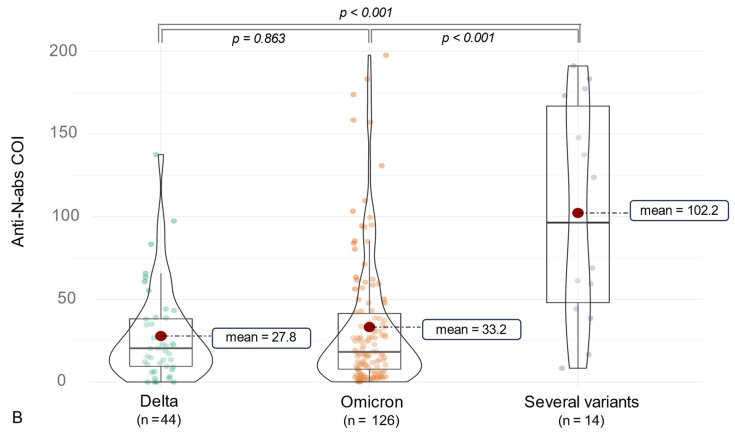
The figure shows violin charts to illustrate the variant-related differences in anti-SARS-CoV-2 antibody production. Mean values are visualized as a red dot. (**A**) shows the anti-S/RBD abs for the different variant cohorts. (**B**) illustrates this comparison for the anti-N abs. The count of subjects labeled as “several variants” varied due to limited material, as one subject had available material only for anti-S/RBD1 dilutions.

Considering SARS-CoV-2 ab detection, anti-RBD1 abs were assessed in 100% of the participants, and anti-N abs were measured in 96.2% (*n* = 185). The median anti-N-ab titer was 21.12 [9.07, 45.12] COI, and the median anti-RBD/S-ab level was 21,247.00 [11,494.00, 36,538.00] U/mL (Table 2). Furthermore, variant-associated differences could be observed. Participants infected with the Delta variant (*n* = 44) had a median anti-RBD1/S-ab titer of 16,064.50 [9669.50, 30,972.25] U/mL, and subjects suffering from an Omicron (*n* = 126) infection showed a higher median anti-RBD1/S-ab level of 22,387.50 [14,535.50, 39,342.25] U/mL (*p* = 0.021). Moreover, 15 individuals who might be infected with several SARS-CoV-2 variants, as indicated by epidemiological data, presented a median titer of 10,812.00 [7219.50, 21,182.00] of anti-RBD1/S abs. Of these, most were infected at the time of the Delta spread combined with an Omicron infection (*n* = 10), followed by the combination of Alpha/Omicron (*n* = 3) and Alpha/Delta infections (*n* = 2). Concerning anti-N abs, participants infected by several SARS-CoV-2 variants showed a higher median titer (96.39 [48.03, 166.85] COI) compared to Delta (20.44 [9.49, 38.21] COI) and Omicron (18.23 [7.78, 41.47] COI) infections (*p* < 0.001) (see Figure 3). In addition to 15 participants with two infections of different variants, 4 participants experienced dual infections with the Omicron variant. All of these participants received their first vaccination prior to their second infection, with four individuals experiencing their initial COVID-19 infection before the first vaccination (three individuals infected with the Alpha variant and one individual infected with the Delta variant). A summary of the impact on anti-SARS-CoV-2 antibody formation in the case of multiple SARS-CoV-2 infections is presented in Table 3 and Table 4.

Reflecting significant differences in anti-SARS-CoV-2 antibodies of the Delta and Omicron cohorts, the time of blood sampling had to be addressed. There was a significantly longer period between SARS-CoV-2 infection and sample collection in subjects infected with the Delta variant (mean 152.91 +/− 21.58 days) compared to Omicron (mean 84.48 +/− 22.15 days, *p* < 0.001). Regarding a previous study addressing pure infection-related antibody decline more than one year after COVID-19, this could be assigned to the t2 (95 +/− 43 days after infection) and t3 appointments (157 +/− 50 days after infection). We observed no significant anti-N- and no anti-RBD/S-ab decline between the t2 and t3 appointments [15]. 

Considering the observation that infections involving multiple variants are associated with higher anti-N antibody levels, we conducted a thorough investigation into the interplay of different variants in terms of antibody production. Among 19 participants who experienced multiple infections, the majority exhibited a combination of Delta and Omicron infections (*n* = 10). The cohorts of Alpha and Delta (*n* = 2) and Alpha and Omicron (*n* = 3) were marginal. Additionally, there was a small subcohort of individuals who were infected twice with the Omicron variant (*n* = 4). It is important to note that this comparison serves only as an overview due to the small sample size.

As indicated in Table 3, initially, significantly higher anti-N antibody levels were observed in the combination of Delta and Omicron infection compared to the double Omicron infection. However, after examining these significances using the Benjamini–Hochberg method, the comparison no longer remained statistically significant. Furthermore, in terms of anti-S/RBD antibodies, no significant differences were detected when considering adjusted *p*-values. In addition to comparing individual combinations, we deemed it relevant to contrast the comparison of multiple infections with a single Omicron or Delta infection, and thus, we present this in Table 4. Following multiple testing corrections, a significantly higher level of anti-N antibodies was evident in individuals with a combination of Delta and Omicron infections compared to those with a singular infection of Delta or Omicron (*p*-values = 0.016). The impact on anti-S/RBD1 antibodies can be observed in Table 4. Moreover, we consolidated the subcohorts, depicting individuals with infections of various variants and comparing their SARS-CoV-2 antibody production to that of Delta or Omicron in Figure 3.

Moreover, we assessed COVID-19 symptoms in a structured survey. The variant-associated symptoms are compared in Table 5. Due to the comparison of multiple parameters, we conducted the multiple comparison correction using the Benjamini–Hochberg method to reduce false-positive results. As a result, only anosmia and ageusia (*p*-values = 0.005) remained as significantly more frequent symptoms in Delta infections and sore throat in Omicron infections (*p* = 0.005).

**Figure 4:**
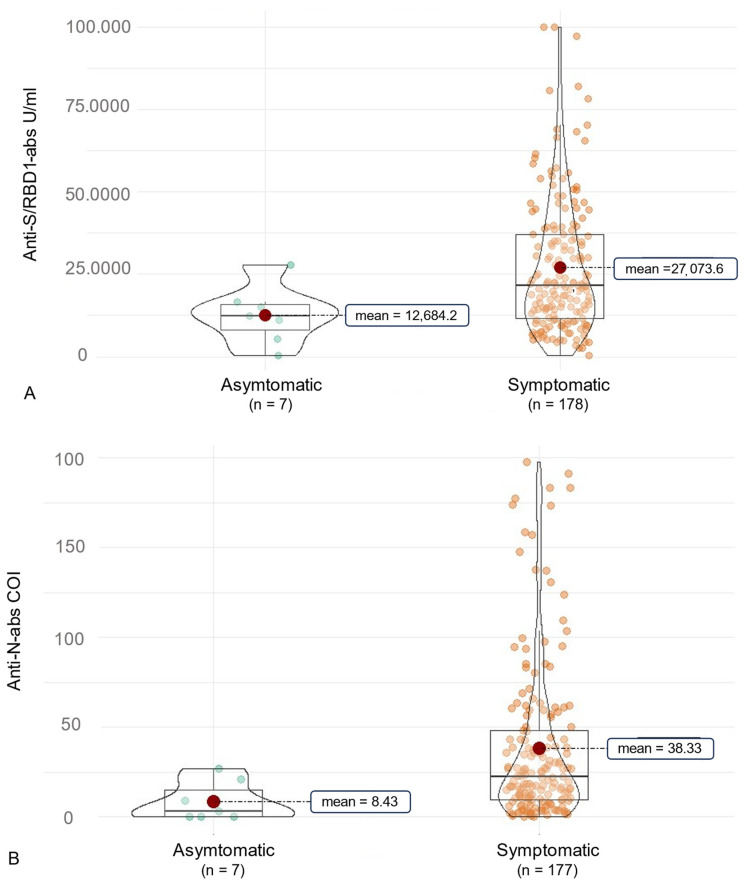
Overall clinical association of SARS-CoV-2 antibodies in individuals infected with the Delta or the Omicron variant. The figure shows violin charts to illustrate the symptom-based differences in anti-SARS-CoV-2 antibody production. Mean values are visualized as a red dot. (**A**) shows the anti-S/RBD abs for individuals experiencing symptomatic or asymptomatic COVID-19. (**B**) illustrates this comparison for the anti-N abs. COI, cut-off index.

**Figure 5:**
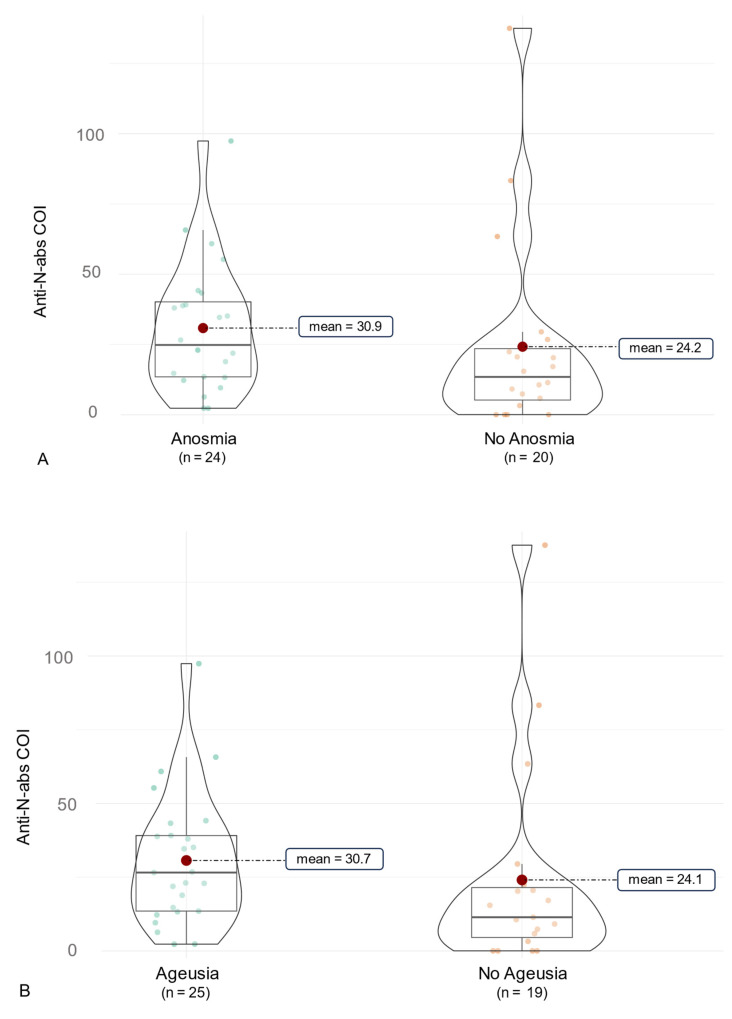
Clinical association of anti-SARS-CoV-2-anti-N antibodies in Delta infections. The figure shows violin charts to illustrate the symptom-based differences in anti-SARS-CoV-2-anti-N antibody production in Delta infections. Mean values are visualized in the violin chart as a red dot. (**A**) anti-SARS-CoV-2-anti-N antibodies for participants with and without Anosmia are illustrated. (**B**) anti-SARS-CoV-2-anti-N antibodies for participants with and without Ageusia are illustrated.

In this context, we examined a correlation of antibody production and COVID-19 symptoms (Figure 4). Initially, symptoms like cough, throat pain, dyspnea, and diarrhea were associated with significantly higher anti-RBD/S abs (*p* < 0.05, Table 6). This was reduced to non-significant implications for throat pain by use of the Benjamini–Hochberg correction (*p* = 0.080). In terms of overall anti-N abs, no significant differences associated with certain symptoms were observed (Table 7). As the symptom of throat pain was reported significantly more often by participants infected with the Omicron variant, we further examined the association of symptoms and SARS-CoV-2-ab production for each variant. In this context, the symptoms diarrhea, cough and myalgia were slightly non-significantly associated with a higher anti-RBD/S-ab production in participants infected with the Omicron variant compared to those without these symptoms (*p* = 0.058, 0.061, 0.093). However, it should be noted that only six subjects of the overall cohort suffered from diarrhea during COVID-19. Furthermore, individuals suffering from throat pain had significantly higher anti-RBD/S-abs (*p* = 0.007) in the Omicron cohort. Nevertheless, there was no significant difference in anti-RBD/S abs in subjects affected by myalgia, cough, throat pain, and diarrhea in the Delta cohort. However, participants of the Delta cohort suffering from ageusia and anosmia had significantly higher anti-N abs compared to those without these symptoms (*p* = 0.050 and *p* = 0.034, respectively, Figure 5). In addition, there was a tendency for higher anti-N abs in Delta-infected patients reporting loss of concentration (*p* = 0.044). 

## 4. Discussion

In this study, we investigated the influence of clinical factors on the humoral immune response in vaccinated individuals infected with two different SARS-CoV-2 variants and we identified some symptoms described in more detail in the following that may be associated with higher antibody production compared to individuals without these symptoms. Furthermore, we tried to obtain a representative overview of the severity of a SARS-CoV-2 infection in German healthcare workers after vaccination examining a structured history of COVID-19 symptoms. This is particularly relevant as the high infection and hospitalization rates of the first COVID-19 waves challenged the healthcare system globally and a reduction in severe COVID-19 cases is of general interest in terms of the success of the vaccination strategy.

Several studies have already demonstrated a reduction in the severity of COVID-19 as a result of SARS-CoV-2 vaccination [16,17]. For example, Vasileiou et al. utilized surveillance data from 99% of the Scottish population to assess the impact of a single vaccine dose on hospitalization. Depending on the vaccine (BNT162b2 and ChAdOx1), they observed a positive vaccine effect on hospitalization ranging from 88% to 91% approximately one month after vaccination [18]. A further study comparing the severity of COVID-19 in healthcare workers after single or double vaccination with unvaccinated individuals, demonstrated milder courses and no hospitalization of vaccinated participants during the observation period [19]. A study by Tenforde et al. also demonstrated a reduction in hospitalization in a comparable age cohort. Overall, across an extended age group, 88.1% of all hospitalized COVID-19 patients were unvaccinated and 11.9% were vaccinated [20]. Vaccination success based on a low hospitalization rate was also supported in our study, as 99.5% of the participants underwent ambulatory treatment. This is particularly relevant as our study equally included individuals from the healthcare sector. Nevertheless, the cohort of individuals infected with the Delta variant, a variant associated with more severe cases [21,22], was significantly smaller. This can probably be explained by the higher contagiousness of the Omicron variant. However, Duong et al. described a milder clinical course despite higher contagiousness. They systematically compared differences between the Delta and the Omicron variants and described a 100-fold higher infectivity of the Omicron compared to the Delta variant based on observations in the UK. Despite high overall transmission, a reduction to one-third in terms of hospitalization was reported [23]. 

As increased transmissibility but reduced severity has been confirmed in additional studies [12,21,22,24,25,26], we consequently conducted a more detailed comparison of symptom characteristics for the different variants. Subjects infected with the Omicron variant were significantly more likely to have the symptom of sore throat. In contrast, participants in the Delta cohort were significantly more likely to suffer from anosmia and loss of taste. Tendencies for these differences have equally been reported by Ekroth et al. who observed a higher prevalence of sore throat in individuals infected with the Omicron variant and loss of smell and taste in Delta infections. We could underline these findings with our results. In summary, our evaluation of disease severity based on hospitalization revealed predominantly mild outcomes among vaccinated participants (study aim I). Nevertheless, variant-specific symptoms were also documented. 

Therefore, the inquiry arose as to whether specific symptoms inherently display an association with the humoral immune response. In this regard, a noteworthy elevation in anti-S/RBD abs was observed in cases of the Omicron variant, specifically when individuals manifested the symptom of a sore throat. The association with diarrhea was viewed with skepticism due to the limited sample size and warrants further investigation in subsequent studies. This led us back to our primary research inquiry, exploring whether vaccinations contribute to less severe outcomes and, consequently, a diminished manifestation of symptoms specific to the disease and antibody production. Overall, the levels of anti-N abs were comparatively lower compared to our previous study examining unvaccinated individuals infected with the Alpha variant, supporting the primary study hypothesis [15]. Additionally, higher levels of anti-N abs were present in cases with typical Delta symptoms compared to individuals not suffering from ageusia or anosmia, further endorsing the hypothesis of a clinic–antibody association. Beyond symptom–antibody correlations, we also observed variant-related differences in the production of antibodies. The anti-S/RBD abs were significantly higher in the Omicron cohort than in the Delta cohort with no difference in anti-N-ab production (study aims II and III). 

Nevertheless, a few limitations need to be considered in this context. One aspect to address is the fact that the Delta variant occurred earlier than the Omicron variant, creating a significant time gap between infection and sample collection, which could act as a confounder for variant-specific differences in antibody levels. At least, concerning our prior research, there was no indication of a significant decline in anti-S/RBD or anti-N abs in a comparable period after infection [15]. Moreover, different vaccine combinations might affect the anti-RBD/S-ab level as well, revealing a further relevant influencing aspect. In this context, the infection with several variants displays another antibody production affecting aspect. However, we were able to demonstrate through an examination of various subcohorts of multiply affected individuals that a combination of Delta and Omicron infection is associated with higher disease-specific anti-N antibody levels (*p* = 0.016). This highlights that the influence on antibody production is multifactorial, which must be critically considered when assessing a symptom–antibody correlation. Furthermore, in addition to the influence exerted by clinical factors, SARS-CoV-2 variants, and previous vaccinations, the potential impact of antiviral therapy with immunoglobulins or monoclonal antibodies is another significant aspect that may affect antibody detection in the individuals under consideration. Kim et al. demonstrated, among others, that COVID-19 therapy with monoclonal antibody treatment was associated specifically with lower anti-spike IgM antibody titers but interestingly did not affect anti-nucleocapsid antibodies [27]. Because we did not explicitly inquire about this, a definitive exclusion of any related bias remains inconclusive and must be critically considered. In future follow-up studies, such aspects should be systematically addressed. A further limitation is the fact that our first intention was to compare the overall manifestation of symptoms with asymptomatic courses concerning the development of disease-specific anti-N abs. However, our study only enrolled a limited number of asymptomatic individuals. 

Furthermore, T-cell-mediated immune responses constitute an essential aspect of the immune defense against intracellular pathogens. Notably, T-cell responses have been identified as pivotal for the elimination of viruses such as influenza viruses and SARS-CoV during infection [28,29]. Importantly, the cellular immune response exhibits greater resilience against viral mutations, with a minimal impact from single amino acid substitutions. Therefore, more investigation of cellular immune responses is warranted, particularly in the context of vaccinated individuals lacking detectable antibodies [30]. However, because no such scenarios were observed in the study cohort, a detailed analysis of cellular immunity was not conducted. Moreover, previous studies described that protective immunity mediated by antibodies with a virus-neutralizing capacity correlates with anti-RBD/S1 antibodies [31,32,33]. Thus, the anti-RBD/S1-pan-Ig serves as a surrogate parameter to monitor humoral SARS-CoV-2 immunity in our study. Finally, some of these limitations were explicitly addressed and we conducted a thorough examination of the points by analyzing the existing clinical data. 

In summary, our findings may indicate variant-specific differences in antibody production, suggesting potentially higher anti-S/RBD abs in Omicron variant infections and higher anti-N abs in individuals infected with several variants (study aim II). This might be due to a longer or more intense exposition to the virus leading to a higher serological immune response regarding anti-N abs. As most participants in this cohort were infected by the Delta and Omicron variants, the dual stimulation in a very short time interval might have potentially intensified the antibody production. However, this would need to be verified in subsequent studies with a larger cohort specifically addressing this aspect. Furthermore, a valuable aspect to investigate would be whether higher anti-S/RBD antibody levels are attributed to variant-specific mutations. For instance, Da Costa et al. described the mutations N440K, T478K, Q493R, and Q498R on RBD in the spike protein of the Omicron variant that could underline this aspect [34]. These mutations could conceivably trigger a higher immune response, an aspect that needs further investigation. Nevertheless, some participants infected with the Delta cohort equally exhibited high anti-SARS-CoV-2 values (for example, 80.676 U/mL). Thus, this implies that there might be more influencing factors such as the infection-related clinic. Nevertheless, it must be considered that there is an influence on the RBD/S antibodies due to prior vaccination. Therefore, in this paper, we want to focus primarily on the anti-N abs not affected by the vaccination. In this context, we emphasize particularly in the case of an infection with the Delta variant that the presence of ageusia and anosmia is associated with higher anti-N abs compared to those not exhibiting these symptoms.

Summarizing our study population, individuals without symptoms exhibited diminished levels of anti-SARS-CoV-2 antibodies (study aim III). In this context, we observed a clinical correlation with disease-specific antibodies in individuals infected with the Delta variant, highlighting that higher anti-N antibodies were associated with the typical symptoms of ageusia and anosmia compared to those without. Notably, the predominant observation in vaccinated individuals was milder disease courses with outpatient management (aim I). This overall reduction in clinical severity is associated with a slightly decreased production of disease-specific anti-N antibodies compared to pre-vaccination infection data [15]. 

An intriguing avenue for further research could be the exploration of a post-COVID-19 syndrome with cognitive symptoms in vaccinated subjects infected with different SARS-CoV-2 variants. In our study, variant-specific differences emerged, showing non-significant slight indications for a higher tendency of such symptoms during the acute phase of COVID-19 in our Omicron cohort. Yuan et al. also observed similar indications. They quantified cognitive symptoms using the Mini-Mental State Examination (MMSE) and Montreal Cognitive Assessment (MoCA), which urgently needs to be supplemented in follow-up analyses [35]. Overall, the relevance and implications for the occurrence of post-COVID-19 syndrome in those cohorts should be illuminated in subsequent studies, as this aspect has the potential to longitudinally impact the lives of those affected.

## 5. Conclusions

In conclusion, our study reveals variant-specific variations in antibody production and COVID-19 symptoms. Despite this, vaccinated individuals infected with Omicron or Delta exhibit overall mild disease courses. Moreover, asymptomatic individuals manifest lower anti-SARS-CoV-2 antibody levels, suggesting a clinical correlation between disease-specific antibodies and distinctive symptoms. Furthermore, it is imperative to delve deeper into investigating post-COVID-19 syndrome regarding cognitive complaints in individuals infected with the Omicron variant in subsequent studies.

## Figures and Tables

**Table 1:** Demographic, clinical characteristics, medical history, and outcome.

Variable	All Subjects (*n =* 185)	
Demographics		
Sex F/M (%)	142/43 (76.8/23.2)	
Age (mean (SD))	36.04 (11.65)	
BMI (median [IQR])	23.66 [21.72, 27.85]	
Smoking (%)	28 (15.1)	
Medical profession (%)	184 (99.5)	
Patient contact		
Working in intensive care unit (%)	35 (18.9)	
Working in a normal care unit (%)	66 (35.7)	
Rare patient contact (%)	27 (14.6)	
No patient contact (%)	57 (30.8)	
Medical history		
Lung disease (%)	18 (9.7)	
Autoimmune disease (%)	17 (9.2)	
Immunosuppression (%)	3 (33.3)	
First vaccination dose		
BioNTech/Pfizer (%)	120 (64.9)	
Moderna (%)	16 (8.6)	
AstraZeneca (%)	48 (25.9)	
Different (%)	1 (0.5)	
COVID-19 before first vaccination (%)	0/4 (2.16) *	
Days between positive qPCR and first vaccination (median [IQR])	313.00 [250.00, 373.00]	
Second vaccination dose		
BioNTech/Pfizer (%)	155 (84.2)	
Moderna (%)	16 (8.7)	
AstraZeneca (%)	13 (7.1)	
COVID-19 before second vaccination (%)	1/5 (2.7) *	
Days between positive qPCR and second vaccination (mean (SD))	256.42 (88.3)	
Third vaccination dose		
BioNTech/Pfizer (%)	156 (92)	
Moderna (%)	29 (18.0)	
COVID-19 before third vaccination (%)	26/30 (16.2) *	
Positive qPCR before third vaccination (days) (median [IQR])	−92.00 [−103.50, −79.50]	
Positive qPCR after third vaccination (days) (median [IQR])	81.00 [62.00, 105.00]	
Influenza vaccination (%)	76 (41.1)	
Indication for SARS-CoV-2 qPCR *^1^		
Routine Hospital Instructions (%)	37 (20.0)	
Symptomatic (%)	105 (56.8)	
SARS-CoV-2 contact (%)	87 (47.0)	
COVID-19 outpatient care (%)	184 (99.5)	

* Four individuals, whose second infection occurred after the initial vaccination, nonetheless experienced an infection before receiving the first vaccination. *^1^ Multiple answers accepted. Five participants underwent a 4th vaccination with BionTech/Pfizer. The percentage is presented in brackets. SD, Standard Deviation; IQR, Interquartile Range; IQR is presented in square brackets.

**Table 2:** Types of SARS-CoV-2 antibodies.

Anti-SARS-CoV-2 abs	(*n* = 185)
Anti-N (positive) (%)	177 (96.2)
Anti-N (median [IQR])	21.12 [9.07, 45.12]
Anti-RBD/S1 (positive) (%)	185 (100.0)
Anti-RBD/S1 (U/mL) (median [IQR])	21,247.00 [11,494.00, 36,538.00]

The percentage is presented in brackets. IQR, Interquartile Range; IQR is presented in square brackets.

**Table 3:** Influence of preinfections on SARS-CoV-2 antibodies.

	Type of SARS-CoV-2 Variant					
Anti-SARS-CoV-2 abs	Alpha and Delta	Delta and Omicron	Alpha and Omicron	Omicron and Omicron	*p*-Values	BH Correction
*n*	2	10	3	4		
Anti-N abs (median [IQR])	53.75 [46.14, 61.37]	99.22 [48.03, 166.85]			0.519	0.566
Anti-N abs (median [IQR])	53.75 [46.14, 61.37]		153.50 [138.65, 168.35]		0.121	0.219
Anti-N abs (median [IQR])	53.75 [46.14, 61.37]			15.98 [11.91, 16.63]	0.064	0.154
Anti-N abs (median [IQR])		99.22 [48.03, 166.85]	153.50 [138.65, 168.35]		0.390	0.468
Anti-N abs (median [IQR])		99.22 [48.03, 166.85]		15.98 [11.91, 16.63]	0.024	0.154
Anti-N abs (median [IQR])			153.50 [138.65, 168.35]	15.98 [11.91, 16.63]	0.064	0.154
Anti-S/RBD abs (median [IQR])	4682.00 [4493.50, 4870.50]	19,780.00 [11,070.75, 27,964.50]			0.032	0.154
Anti-S/RBD abs (median [IQR])	4682.00 [4493.50, 4870.50]		10,258.00 [7318.00, 10,535.00]		0.248	0.331
Anti-S/RBD abs (median [IQR])	4682.00 [4493.50, 4870.50]			13,251.00 [10,215.50, 28,164.25]	0.064	0.154
Anti-S/RBD abs (median [IQR])		19,780.00 [11,070.75, 27,964.50]	10,258.00 [7318.00, 10,535.00]		0.128	0.219
Anti-S/RBD abs (median [IQR])		19,780.00 [11,070.75, 27,964.50]		13,251.00 [10,215.50, 28,164.25]	0.671	0.671
Anti-S/RBD abs (median [IQR])			10,258.00 [7318.00, 10,535.00]	13,251.00 [10,215.50, 28,164.25]	0.157	0.236

Comparison of non-normally distributed continuous variables was performed with the Kruskal–Wallis test. IQR, Interquartile Range; IQR is presented in square brackets; BH, Benjamini–Hochberg correction.

**Table 4:** Influence of preinfections on SARS-CoV-2 abs compared to single Omicron or Delta infection.

Type of SARS-CoV-2 Variant	Alpha and Delta	Delta and Omicron	Alpha and Omicron	Omicron and Omicron	Delta	Omicron	*p*-Values	BH Correction
*n*	2	10	3	4	44	122		
Anti-N abs (median [IQR])	53.75 [46.14, 61.37]				20.44 [9.49, 38.21]		0.106	0.208
Anti-N abs (median [IQR])		99.22 [48.03, 166.85]			20.44 [9.49, 38.21]		0.002	0.016
Anti-N abs (median [IQR])			153.50 [138.65, 168.35]		20.44 [9.49, 38.21]		0.021	0.061
Anti-N abs (median [IQR])				15.98 [11.91, 16.63]	20.44 [9.49, 38.21]		0.296	0.382
Anti-N abs (median [IQR])	53.75 [46.14, 61.37]					18.80 [7.78, 42.76]	0.153	0.245
Anti-N abs (median [IQR])		99.22 [48.03, 166.85]				18.80 [7.78, 42.76]	0.002	0.016
Anti-N abs (median [IQR])			153.50 [138.65, 168.35]			18.80 [7.78, 42.76]	0.023	0.063
Anti-N abs (median [IQR])				15.98 [11.91, 16.63]		18.80 [7.78, 42.76]	0.278	0.426
Anti-S/RBD abs (median [IQR])	4682.00 [4493.50, 4870.50]				16,064.50 [9669.50, 30,972.25]		0.041	0.090
Anti-S/RBD abs (median [IQR])		19,780.00 [11,070.75, 27,964.50]			16,064.50 [9669.50, 30,972.25]		0.824	0.906
Anti-S/RBD abs (median [IQR])			10,258.00 [7318.00, 10,535.00]		16,064.50 [9669.50, 30,972.25]		0.117	0.214
Anti-S/RBD abs (median [IQR])				13,251.00 [10,215.50, 28,164.25]	16,064.50 [9669.50, 30,972.25]		0.970	0.970
Anti-S/RBD abs (median [IQR])	4682.00 [4493.50, 4870.50]					22,494.00 [14,728.75, 39,342.25]	0.021	0.063
Anti-S/RBD abs (median [IQR])		19,780.00 [11,070.75, 27,964.50]				22,494.00 [14,728.75, 39,342.25]	0.310	0.426
Anti-S/RBD abs (median [IQR])			10,258.00 [7318.00, 10,535.00]			22,494.00 [14,728.75, 39,342.25]	0.021	0.063
Anti-S/RBD abs (median [IQR])				13,251.00 [10,215.50, 28,164.25]		22,494.00 [14,728.75, 39,342.25]	0.381	0.466

Comparison of non-normally distributed continuous variables was performed with the Kruskal–Wallis test. IQR, Interquartile Range; IQR is presented in square brackets; BH Benjamini–Hochberg correction.

**Table 5:** Variant-related clinic of SARS-CoV-2 infections.

Symptom	Delta (*n* = 44) (%)	Omicron (*n* = 126) (%)	*p-*Value	*p-*Value BH Correction
Fever	18 (40.9)	55 (43.7)	0.860	0.983
Night sweats	5 (11.4)	14 (11.1)	1.000	1.000
Myalgia	4 (9.1)	7 (5.6)	0.478	0.695
Headache	32 (72.7)	84 (66.7)	0.573	0.764
Cough	22 (50.0)	84 (66.7)	0.070	0.187
Throat pain	17 (38.6)	86 (68.3)	0.001	0.005
Dyspnea	10 (22.7)	48 (38.1)	0.068	0.187
Common cold	24 (54.5)	86 (68.3)	0.142	0.325
Diarrhea	2 (4.5)	4 (3.2)	0.650	0.800
Nausea	1 (2.3)	5 (4.0)	1.000	1.00
Loss of Appetite	3 (6.8)	5 (4.0)	0.428	0.695
Loss of concentration	5 (11.4)	33 (26.2)	0.057	0.187
Depression	1 (2.3)	1 (0.8)	0.452	0.695
Hair loss	1 (2.3)	0 (0.0)	0.259	0.518
Anosmia	24 (54.5)	21 (16.7)	<0.001	0.005
Ageusia	25 (56.8)	21 (16.7)	<0.001	0.005

Comparison of categorical variables was performed with Fisher’s exact test. The percentage is presented in brackets. BH, Benjamini–Hochberg correction.

**Table 6:** SARS-CoV-2 anti-S/RBD antibodies related to COVID-19 symptoms.

Symptom	Individuals Exhibiting the Symptom Anti-S/RBD abs (Median [IQR])	*n*	Individuals Not Exhibiting the Symptom Anti-S/RBD abs (Median [IQR])	*n*	*p-*Value	*p-*Value BH Correction
Fever	19,438 [11,140; 33,794.]	106	21,861 [12,947; 38,839]	78	0.233	0.414
Night sweats	21,483 [11,494; 35,856]	156	20,975 [12,450; 37,735]	20	0.747	0.750
Myalgia	20,719 [11,138; 35,453]	173	31,884 [20,686; 39,585]	12	0.411	0.658
Headache	20,404 [10,812; 38,307]	61	21,655 [13,276; 35,067]	124	0.725	0.750
Cough	17,099 [10,730; 30,334]	70	22,452 [14,475; 39,675]	115	0.015	0.116
Throat pain	17,468 [10,227; 30,334]	70	22,668 [14,241; 41,173]	115	0.005	0.080
Dyspnea	18,002 [10,812; 35,856]	125	24,217 [15,966; 37,735]	60	0.028	0.116
Common cold	20,719 [10,564; 30,180]	63	22,003 [14,116; 39,179]	112	0.128	0.386
Diarrhea	20,850 [11,141; 35,333]	179	42,252 [35,215; 52,360]	6	0.029	0.116
Nausea	20,975 [11,140; 36,368]	178	33,829 [24,803; 37,419]	7	0.211	0.414
Loss of Appetite	21,483 [11,878; 35,856]	177	12,852 [8688; 43,2201]	8	0.562	0.750
Loss of Concentration	20,225 [11,138; 35,453]	145	24,259 [14,641; 41,187]	40	0.169	0.386
Depression	21,247 [11,319; 36,197]	183	33,899 [23,463; 44,334]	2	0.652	0.750
Hair loss	21,174 [11,407, 36,027]	184	54,769 [54,769; 54,769]	1	0.160	0.386
Anosmia	21,483 [11,494; 38,487]	137	20,452 [11,464; 33,845]	48	0.574	0.750
Ageusia	21,365 [11,124; 38,602]	136	21,100 [11,878; 33,675]	49	0.750	0.750

Comparison of non-normally distributed continuous variables was performed with the Kruskal–Wallis test. IQR, Interquartile Range; IQR is presented in square brackets; BH Benjamini–Hochberg correction.

**Table 7:** SARS-CoV-2 anti-N antibodies related to COVID-19 symptoms.

Symptom	Individuals Exhibiting the Symptom Anti-N abs (Median [IQR])	*n*	Individuals Not Exhibiting the Symptom Anti-N abs (Median [IQR])	*n*	*p-*Value	*p-*Value BH Correction
Fever	19.09 [6.07, 43.27]	106	23.39 [10.58, 54.00]	78	0.278	0.789
Night sweats	20.44 [9.07, 49.96]	156	23.19 [9.85, 35.13]	20	0.528	0.789
Myalgia	20.14 [8.70, 45.14]	173	35.13 [15.83, 41.05]	12	0.263	0.789
Headache	17.59 [4.62, 45.14]	61	22.84 [10.30, 45.07]	124	0.398	0.789
Cough	16.94 [5.88, 48.46]	70	23.72 [10.64, 44.00]	115	0.245	0.789
Throat pain	20.44 [7.74, 43.93]	70	21.78 [9.37, 46.88]	115	0.689	0.789
Dyspnea	22.16 [7.80, 58.85]	125	19.31 [10.62, 37.55]	60	0.690	0.789
Common cold	18.90 [8.16, 32.29]	63	24.79 [9.64, 59.37]	112	0.100	0.789
Diarrhea	20.44 [9.19, 44.23]	179	36.57 [14.73, 45.32]	6	0.632	0.789
Nausea	20.59 [9.28, 44.24]	178	26.79 [7.53, 82.97]	7	0.859	0.883
Loss of Appetite	21.12 [8.38, 48.36]	177	26.68 [15.87, 39.33]	8	0.592	0.789
Loss of concentration	21.91 [9.16, 44.24]	145	16.89 [8.69, 43.14]	40	0.646	0.789
Depression	20.44 [8.89, 44.23]	183	97.55 [67.78, 127.33]	2	0.115	0.789
Hair loss	20.59 [8.98, 46.00]	184	38.01 [38.01, 38.01]	1	0.516	0.789
Anosmia	19.98 [8.37, 50.16]	137	23.08 [11.45, 38.97]	48	0.883	0.883
Ageusia	18.76 [7.80, 49.96]	136	25.65 [13.04, 39.93]	49	0.424	0.789

Comparison of non-normally distributed continuous variables was performed with the Kruskal–Wallis test. IQR, Interquartile Range; IQR is presented in square brackets; BH, Benjamini–Hochberg correction.

## Data Availability

The data presented in this study are available on request from the corresponding author.

## Supplementary Materials

The following supporting information can be downloaded at https://www.mdpi.com/article/10.3390/vaccines12020163/s1, Table S1: Detailed Clinical Data.
