# Supplementary material for: The Impact of Clinical Factors and SARS-CoV-2 Variants on Antibody Production in Vaccinated German Healthcare Professionals Infected Either with the Delta or the Omicron Variant
Source: Vaccines (Basel). 2024 Feb 5;12(2):163. doi: 10.3390/vaccines12020163 (PMC10893335; doi:10.3390/vaccines12020163)
Supplement: Supplementary file 1 [file vaccines-12-00163-s001.zip › vaccines-2799173-supplementary.pdf]

Table S1: Detailed Clinical Data

**COVID-19 symptoms***	**Overall participant** (n = 185)
Fever (%)	79 (42.7)
Night sweat (%)	20 (10.8)
Diarrhea (%)	6 (3.2)
Cough (%)	115 (62.2)
Nausea (%)	7 (3.8)
Loss of appetite (%)	8 (4.3)
Dyspnea (%)	60 (32.4)
Myalgia (%)	12 (6.5)
Concentration deficit (%)	40 (21.6)
Depression (%)	2 (1.1)
Hair loss (%)	1 (0.5)
Anosmia (%)	48 (25.9)
Ageusia (%)	49 (26.5)
Novel Headache (%)	124 (67.0)
Throat pain (%)	115 (62.2)
Common cold (%)	122 (65.9)
Different (%)	126 (68.1)
Further classification of „different"	
Tiredness (%)	32 (17.3)
Exhaustion (%)	45 (24.3)
Limb pain (%)	50 (27.0)
Shivering (%)	17 (9.1)
Respiratory complaints (%)	6 (3.2)
Ear pain (%)	16 (8.6)
Neurological symptoms (%)	6 (3.2)
Further (%)	16 (8.6)
Pain intensity (%)	
1	4 (3.2)
2	15 (12.1)
3	16 (12.9)
4	14 (11.3)
5	19 (15.3)
6	15 (12.1)
7	16 (12.9)
8	19 (15.3)
9	2 (1.6)
10	4 (3.2)
Vaccination reaction*	
Vaccination reaction yes/no (%)	150/35 (80.1/18.9)
Reaction at 1rst vaccination (%)	130 (70.3)
Local reaction (%)	106 (81.4)
Intensity (1 – 5)	
1	83 (79.8)
2	15 (14.4)
3	6 (5.8)

Duration	
1 day	48 (45.7)
2 -3 days	43 (41.0)
> 3 days	14 (13.3)
Systemic reaction (%)	85 (65.4)
Intensity (1 – 5)	
1	34 (40.0)
2	35 (41.2)
3	13 (15.3)
4	2 (2.4)
5	1 (1.2)
Duration	
1 day	38 (44.7)
2 -3 days	36 (42.4)
> 3 days	11 (12.9)
Use of analgesics (%)	33 (17.8)
Ibuprofen	13 (39.4)
Acetaminophen	16 (48.5)
Metamizole	2 (6.1)
Acetylsalicylic acid	1 (3.0)
Gabapentin	1 (3.0)
**Reaction at 2nd vaccination (%)**	122 (65.9)
Local reaction (%)	103 (84.4)
Intensity (1 – 5)	
1	87 (84.5)
2	13 (12.6)
3	3 (2.9)
Duration	
1 day	53 (51.5)
2 -3 days	39 (37.9)
> 3 days	11 (10.7)
Systemic reaction (%)	74 (60.7)
Intensity (1 – 5)	
1	34 (46.6)
2	28 (38.4)
3	9 (12.3)
4	2 (2.7)
5	0 (0.0)
Duration	
1 day	38 (51.4)
2 -3 days	28 (37.8)
> 3 days	8 (10.8)
Use of analgesics (%)	26 (21.3)
Ibuprofen	14 (53.8)
Acetaminophen	11 (42.3)
Gabapentin	1 (3.8)
Reaction at 3rd vaccination (%)	97 (52.4)

Local reaction (%)	83 (85.6)
Intensity (1 – 5)	
1	70 (84.3)
2	7 (8.4)
3	4 (4.8)
4	1 (1.2)
5	1 (1.2)
Duration	
1 day	37 (44.6)
2 -3 days	36 (43.4)
> 3 days	10 (12.0)
Systemic reaction (%)	53 (54.6)
Intensity (1 – 5)	
1	25 (47.2)
2	17 (32.1)
3	9 (17.0)
4	2 (3.8)
5	0 (0.0)
Duration	
1 day	27 (50.9)
2 -3 days	20 (37.7)
> 3 days	6 (11.3)
Use of analgesics (%)	14 (14.4)
Ibuprofen	5 (35.7)
Acetaminophen	7 (50.0)
Metamizole	1 (7.1)
Gabapentin	1 (7.1)
